# Changes in Caregiver Burden Following Cataract Surgery in Older Adults with Moderate-to-Severe Dementia: A Prospective Pilot Study

**DOI:** 10.3390/geriatrics11040086

**Published:** 2026-07-14

**Authors:** Reiko Umeya, Koichi Ono, Yuto Yoshida

**Affiliations:** 1 Department of Ophthalmology, Juntendo University School of Medicine, 3-1-3 Hongo, Bunkyo-ku 113-8431, Tokyo, Japan; 2 Department of Ophthalmology, Juntendo Tokyo Koto Geriatric Medical Center, 3-3-20 Shinsuna, Koto-ku 136-0075, Tokyo, Japan

**Keywords:** dementia, cataract surgery, caregiver burden, Zarit Burden Interview, visual impairment, older adults

## Abstract

**Background:** Dementia is a global public health priority, and visual impairment has been identified as a modifiable risk factor. While cataract surgery improves quality of life in mild cognitive impairment, its impact on the burden of caregivers supporting patients with moderate-to-severe dementia remains underexplored. **Objectives:** To explore changes in caregiver burden following cataract surgery in older adults with dementia. **Methods:** This prospective, single-center, observational pilot study included 28 patient–caregiver pairs aged ≥ 75 years with dementia who underwent cataract surgery. Caregiver burden was assessed using the Japanese version of the Zarit Burden Interview (J-ZBI) at baseline and 3 months postoperatively. Patient outcomes included best-corrected visual acuity (logMAR), Mini-Mental State Examination (MMSE), and the Barthel Index (BI). Pre- and postoperative values were compared using paired *t*-tests, with exploratory analyses to identify factors associated with postoperative burden. **Results:** Visual acuity significantly improved after surgery (*p* < 0.001). The retention rate was 84.8%, indicating feasibility of postoperative follow-up. No detectable short-term changes were observed in J-ZBI (*p* = 0.48), BI (*p* = 0.15), or MMSE (*p* = 0.89). In exploratory analyses, higher preoperative burden and younger caregiver age were associated with higher postoperative burden. **Conclusions:** Cataract surgery significantly improved visual acuity in older adults with moderate-to-severe dementia. However, no detectable short-term changes were observed in caregiver burden, cognitive function, or activities of daily living during the 3-month follow-up period. In exploratory analyses, potentially hypothesis-generating associations were suggested between higher baseline J-ZBI scores, younger caregiver age, and higher postoperative burden. The study nevertheless demonstrated the feasibility of conducting prospective research in this population and provides preliminary data for future studies with larger sample sizes and longer follow-up periods.

## 1. Introduction

Dementia is a growing global public health challenge that places a substantial burden on patients, caregivers, and healthcare systems. In aging societies, the strain on informal caregivers has reached critical levels, as the symptoms of dementia disrupt daily living and reduce the quality of life (QoL) for both the patient and their families [1,2]. Among the various factors contributing to this burden, sensory impairments—particularly vision loss—have attracted increasing attention [3]. Visual impairment can exacerbate functional decline, potentially accelerate dementia progression [4], and significantly increase caregiver strain [2].

Cataract, the leading cause of reversible visual impairment in older adults, can be effectively treated through surgery. In individuals without cognitive impairment, cataract surgery is a well-established approach for restoring visual acuity and improve activities of daily living and overall QoL [5,6]. More recently, emerging evidence suggests that cataract surgery may also help maintain or improve cognitive performance in patients with mild cognitive impairment (MCI) [7].

However, despite these reported benefits, the effects of cataract surgery in patients with established dementia remain poorly understood. A scoping review by Dawes et al. highlighted that evidence regarding the impact of sensory interventions on caregiver burden remains limited and inconsistent [8]. While Lerner et al. suggested improvements in caregiver distress in a controlled study, their findings were only reported in conference proceedings and involved patients with relatively mild cognitive impairment (mean MMSE 18.3) [8,9]. Furthermore, Sano et al. recently demonstrated functional improvements following cataract surgery in patients with severe dementia in Japan; however, their study was retrospective and focused on highly selected cases requiring general anesthesia [10].

This lack of robust evidence is particularly important in light of the 2024 update of the Lancet Commission on dementia prevention, intervention, and care, which identified untreated vision loss as a modifiable risk factor for dementia [4]. In addition, recent evidence has highlighted the association between visual impairment and postoperative neurocognitive complications. Koga et al. reported that poor preoperative visual acuity was an independent risk factor for postoperative delirium following ophthalmic surgery, particularly among patients with comorbid dementia [11].

Although these findings underscore the importance of visual health in dementia care, the question of how vision restoration influences the lived experience of patients with established dementia—particularly from the caregiver perspective—remains unresolved. Moreover, conducting interventional studies in this population presents substantial ethical and practical challenges, including difficulties in establishing appropriate control groups. Vision is the dominant sensory modality through which humans acquire information from their environment. Visual impairment can reduce orientation, communication, mobility, and independence in activities of daily living, thereby increasing reliance on caregivers. Accordingly, restoration of vision through cataract surgery could theoretically lessen caregiver burden. However, in patients with moderate-to-severe dementia, caregiver burden is multifactorial and may also be influenced by cognitive impairment, behavioral symptoms, physical dependency, and other caregiver-related factors. Therefore, the extent to which improved visual function translates into reduced caregiver burden remains uncertain. Unlike previous studies that primarily focused on visual outcomes in patients with mild cognitive impairment or were based on retrospective surgical series, the present prospective pilot study specifically examined caregiver burden as a primary outcome in older adults with moderate-to-severe dementia. By focusing on this population with established dementia, this study addresses an important gap in the literature and provides pilot data to inform the design of future definitive interventional studies, including considerations regarding sample size estimation and optimal evaluation periods.

Therefore, this prospective pilot study aimed to evaluate the feasibility of conducting clinical research in older adults with moderate-to-severe dementia undergoing cataract surgery, including retention and follow-up rates, and to explore the preliminary impact of surgery on caregiver burden using the Japanese version of the Zarit Burden Interview (J-ZBI).

## 2. Methods

This study was designed as a prospective, single-center, observational study conducted at Juntendo Tokyo Koto Geriatric Medical Center (JTKGMC). All procedures were conducted in accordance with the Declaration of Helsinki and were approved by the Ethics Committee of JTKGMC (approval number: G20-0034).

### 2.1. Study Design and Participants

**Figure 1:**
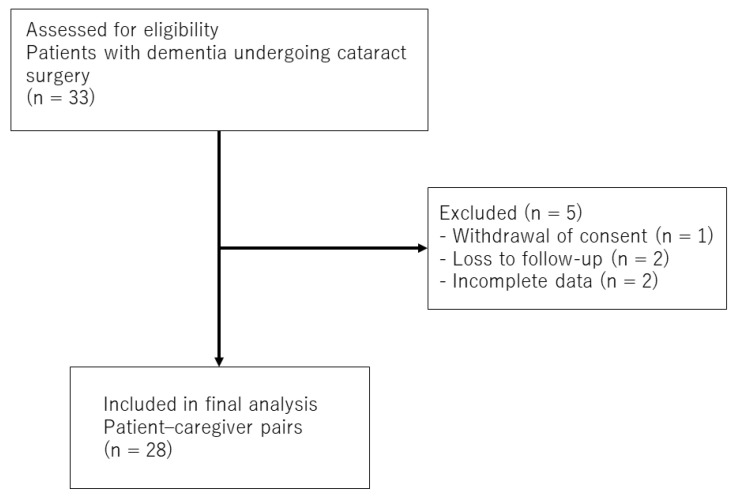
Flow diagram of patient–caregiver enrollment. Flow diagram illustrating the prospective enrollment, exclusion, and final inclusion of patient–caregiver pairs in this study.

Consecutive older patients (≥75 years) with a clinical diagnosis of dementia who underwent cataract surgery were recruited between 1 April 2021, and 31 March 2025. Eligible patients had received a formal diagnosis of dementia from a specialist (geriatrician, neurologist, or dementia specialist) prior to referral. Patients whose cognitive impairment was considered limited to mild cognitive impairment (MCI) were not eligible for inclusion. Consistent with these eligibility criteria, the study population had a mean MMSE score of 13.1 ± 5.6, reflecting predominantly moderate-to-severe cognitive impairment. All participants lived with family members or caregivers and were not living alone at the time of enrollment. A total of 33 patient–caregiver pairs were enrolled. Exclusion criteria included the presence of severe ocular comorbidities other than cataract or refusal to participate. When patients were judged to have limited capacity to provide informed consent, consent was obtained from their primary caregivers. Written informed consent was obtained from caregivers. In addition, assent was obtained from patients whenever possible. The patient selection and enrollment process is summarized in Figure 1.

### 2.2. Assessments and Outcome Measures

Caregiver burden was assessed using the Zarit Burden Interview (ZBI), a widely used 22-item instrument designed to evaluate the physical, emotional, and psychological impact of caregiving [12]. Each item is rated on a 5-point Likert scale (0–4), yielding a total score ranging from 0 to 88, with higher scores indicating greater caregiver burden.

In the present study, J-ZBI was used. The J-ZBI has been validated for use in Japanese populations and has demonstrated good reliability and construct validity among caregivers of older adults, including those caring for patients with dementia [13]. The questionnaire was completed by the primary caregiver at baseline (preoperatively) and again at 3 months postoperatively. To ensure consistency and minimize information bias, caregiver-reported outcome measures were completed by the same primary caregiver at baseline and at the 3-month follow-up. Clinical data collected from patients included best-corrected visual acuity (BCVA), which was converted to the logMAR for analysis. To reflect patients’ overall functional visual status in daily life, the BCVA of the better-seeing eye was used for all visual acuity analyses at both baseline and follow-up.

In addition, because all participants had dementia and lived with caregivers, postoperative care, including eye-drop administration and attendance at follow-up visits, was supported by caregivers as needed. Cognitive function was assessed using the MMSE [14], and functional status was evaluated with the BI [15] for activities of daily living. Demographic variables such as patient age, sex were also recorded. The primary outcome was the change in caregiver burden (J-ZBI score) from baseline to 3 months postoperatively. The secondary outcome was the association of postoperative J-ZBI with various patient and caregiver characteristics.

### 2.3. Statistical Analysis

Pre- and postoperative values for J-ZBI, BCVA, BI, and MMSE were compared using paired *t*-tests. Given the relatively small sample size, Wilcoxon signed-rank tests were additionally performed as a sensitivity analysis to assess the robustness of the findings. To explore factors associated with postoperative caregiver burden, univariate analyses were performed. Patient sex was included as a categorical variable and compared using independent-samples *t*-tests. Continuous variables, including preoperative J-ZBI score, caregiver age, patient age, MMSE score, and BI score, were examined using simple linear regression analyses.

Given the pilot nature of the study and the limited sample size (*n* = 28), multivariable analysis was performed as an exploratory approach to identify potential factors associated with postoperative caregiver burden. To minimize the risk of overfitting, the number of independent variables was restricted to those with a *p*-value < 0.10 in univariate analyses and a stepwise selection procedure was applied. This analysis was intended for hypothesis generation rather than definitive causal inference.

Statistical significance was defined as a two-sided *p*-value < 0.05. All statistical analyses were performed using EZR (version 1.63; Jichi Medical University Saitama Medical Center, Saitama, Japan) [16].

## 3. Results

This section presents the baseline characteristics of the study participants, the comparison of clinical outcomes before and after surgery, and an analysis of factors associated with postoperative caregiver burden.

### 3.1. Participant Characteristics

The final analysis included 28 patients–caregiver pairs. Of these, 5 pairs (15.2%) did not complete follow-up due to withdrawal of consent (*n* = 1), loss to follow-up (*n* = 2), or incomplete questionnaire data (*n* = 2). Consequently, 28 pairs (84.8%) completed the 3-month follow-up and were included in the final analysis. The mean age of the patients was 85.5 ± 5.7 years, with a predominance of female patients (21/28, 75.0%). The mean preoperative MMSE score was 13.1 ± 5.6, and the mean BI was 72.3 ± 20.3, indicating a study population with moderate-to-severe cognitive impairment and partial dependence in activities of daily living. Of the 28 patients included in the analysis, 10 (35.7%) underwent surgery under general anesthesia and 18 (64.3%) under local anesthesia. Regarding surgical laterality, 22 patients (78.6%) underwent bilateral cataract surgery and 6 (21.4%) underwent unilateral surgery. No major intraoperative or postoperative complications were observed during the study period. The inclusion of general anesthesia cases reflects the fact that the study participants had severe cognitive impairment.

The mean age of primary caregivers was 59.0 ± 12.3 years. Regarding the caregiver–patient relationship, most caregivers were adult children (21/28, 75.0%), followed by spouses (6/28, 21.4%) and one grandchild (1/28, 3.6%). Detailed baseline characteristics of both patients and caregivers are summarized in Table 1.

### 3.2. Changes in Clinical and Caregiver Outcomes

**Figure 2:**
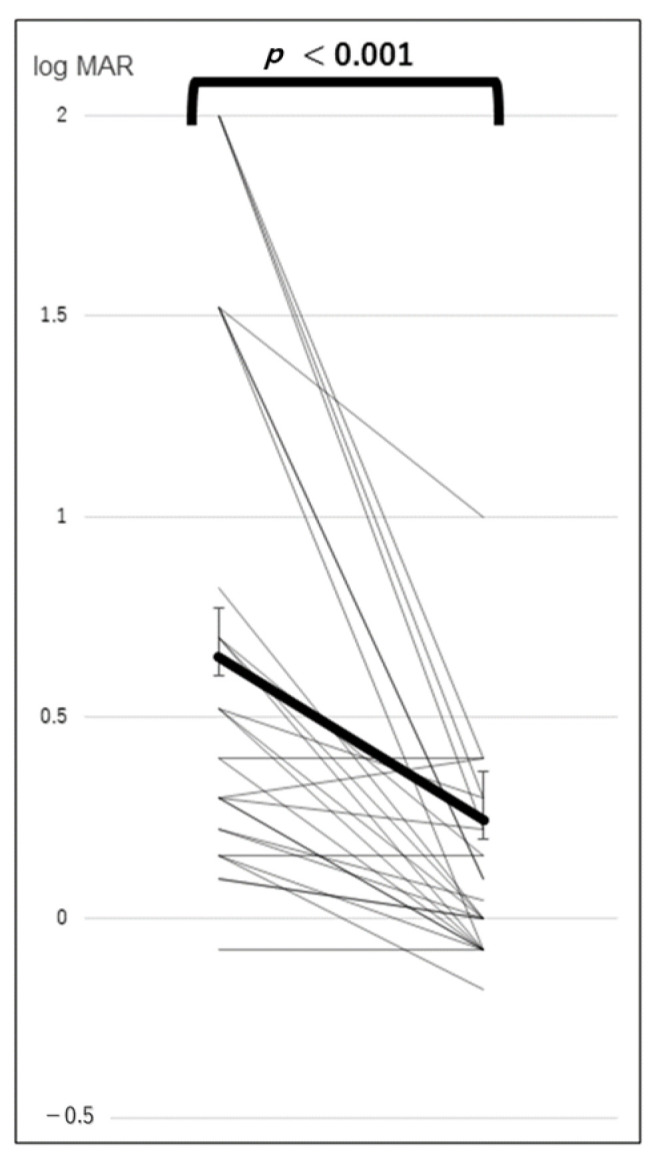
Changes in best-corrected visual acuity before and after cataract surgery. Changes in BCVA (logMAR) at baseline (preoperative) and 3 months after cataract surgery. Bold lines indicate the mean changes. *p* < 0.001.

At the 3-month postoperative follow-up, patients demonstrated a statistically significant improvement in best-corrected visual acuity, with mean logMAR values improving from 0.70 ± 0.65 at baseline to 0.11 ± 0.24 postoperatively (*p* < 0.001) (Figure 2).

**Figure 3:**
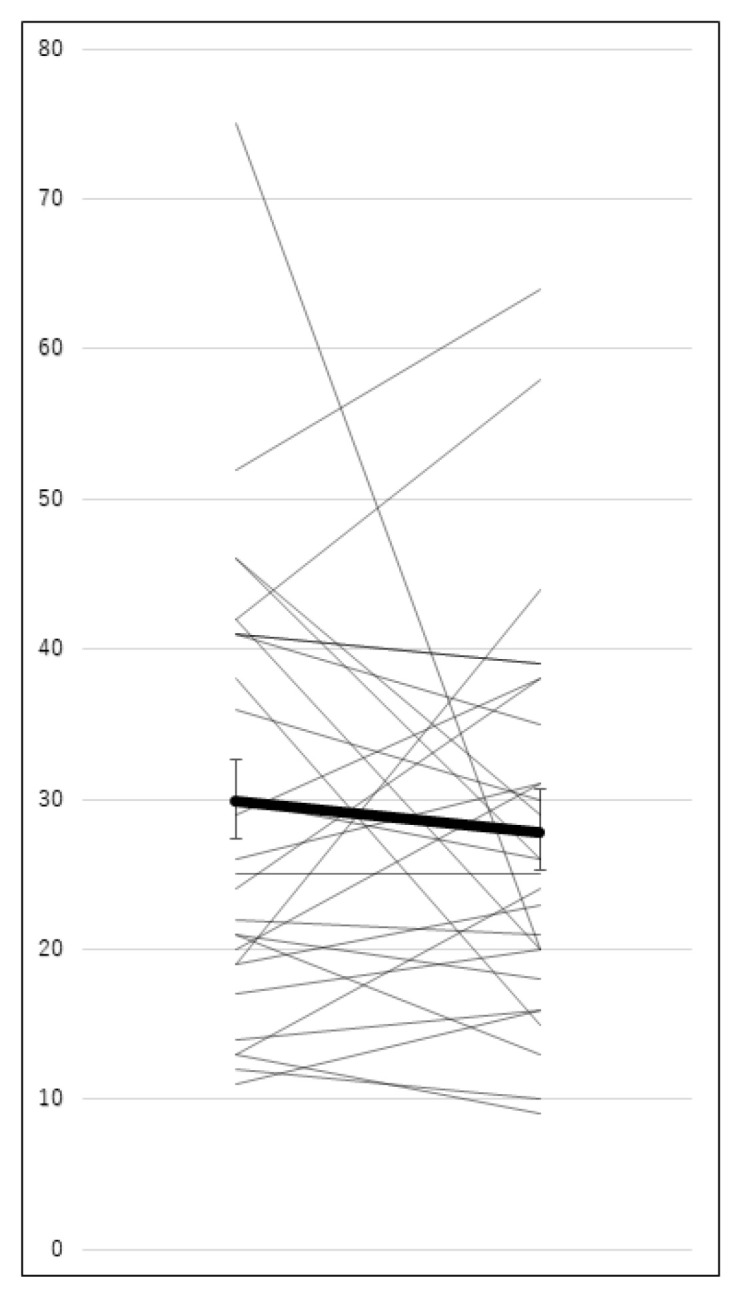
Changes in caregiver burden before and after cataract surgery. Changes in caregiver burden measured by the J-ZBI at baseline (preoperative) and 3 months after cataract surgery. Bold lines indicate the mean changes.

In contrast, no detectable short-term change was observed in caregiver burden, with J-ZBI scores remaining relatively stable (preoperative: 29.9 ± 15.0; postoperative: 27.8 ± 13.2; *p* = 0.48) (Figure 3). Similarly, no detectable short-term changes were observed in patients’ functional independence, as assessed by the BI (72.3 ± 20.3 vs. 75.2 ± 18.6; *p* = 0.15), or in cognitive function, as measured by the MMSE (13.1 ± 5.6 vs. 13.2 ± 6.2; *p* = 0.89). These changes are summarized in Table 2. Sensitivity analyses using the Wilcoxon signed-rank test yielded results consistent with those obtained using the paired *t*-tests for all outcomes, with significant improvement observed only in visual acuity. Values are presented as the mean ± standard deviation. BCVA was converted to logMAR units for analysis. *p*-values were calculated using paired *t*-tests.

### 3.3. Exploratory Factors Associated with Postoperative Caregiver Burden

Univariate analyses were conducted to identify factors potentially associated with postoperative caregiver burden, as measured by the J-ZBI score at 3 months postoperatively. Variables with a *p*-value < 0.10 in univariate analyses were considered candidates for the multivariate linear regression model. Given the pilot nature of the study and the limited sample size, the number of independent variables was restricted to minimize overfitting, and a stepwise selection procedure was applied.

As shown in Table 3, a higher preoperative J-ZBI score was significantly associated with a higher postoperative J-ZBI score in the univariate analysis (*p* = 0.03). Caregiver age and preoperative visual acuity showed trends toward an association with postoperative caregiver burden but did not reach statistical significance. In the exploratory multivariate linear regression analysis, higher preoperative J-ZBI scores and younger caregiver age were independently associated with higher postoperative caregiver burden. Specifically, a higher preoperative J-ZBI score remained significantly associated with a higher postoperative J-ZBI score (standardized β = 0.44, *p* = 0.008), whereas older caregiver age was independently associated with lower postoperative burden (standardized β = −0.38, *p* = 0.047). These findings should be interpreted with caution and considered hypothesis-generating rather than confirmatory given the exploratory nature of the analysis and the limited sample size.

## 4. Discussion

This prospective pilot study provides important preliminary insights into the effects of cataract surgery in older patients with dementia. The main finding was that, although cataract surgery successfully improved visual acuity, this did not lead to a statistically significant reduction in caregiver burden or measurable improvements in patients’ functional independence or cognitive performance over the 3-month follow-up period. These findings suggest that restoration of visual function alone may not be sufficient to produce measurable short-term changes in caregiver burden among patients with established dementia. However, the relatively short follow-up period may have limited the ability to detect longer-term cumulative changes, and these findings should therefore be interpreted with caution. Because caregiver burden reflects cumulative caregiving experiences, the potential long-term effects of visual improvement cannot be excluded, and a longer follow-up period may be required to detect meaningful changes in caregiver burden.

Unlike most previous studies that evaluated visual acuity, activities of daily living, or cognitive outcomes from the patient’s perspective, the present study adopted caregiver burden as the primary outcome measure. By using the Japanese version of the Zarit Burden Interview (J-ZBI), we sought to assess whether visual restoration might influence the caregiving experience itself, an aspect that has received relatively little attention in the ophthalmic literature.

This caregiver-centered approach distinguishes the present study from previous ophthalmic studies and represents a novel contribution to the growing field of sensory interventions in dementia care.

The lack of a significant effect on caregiver burden is likely attributable to the clinical profile of the study population. The patients had a mean MMSE score of 13.1, indicating moderate-to-severe dementia. At this stage, the profound cognitive and functional impairments intrinsic to dementia itself may be too advanced to be meaningfully modified by improvements in visual acuity alone.

The ZBI primarily captures the cumulative physical, emotional, and psychological strain experienced by caregivers over time. It reflects the overall caregiving experience rather than short-term fluctuations in caregiving demands [12,17]. It should also be noted that commonly used cut-off values for the ZBI, such as scores around 24–25 indicating moderate caregiver burden, are primarily intended for clinical screening rather than for detecting short-term longitudinal changes [18]. Therefore, the J-ZBI may have limited sensitivity for detecting subtle short-term changes in caregiver burden following a single intervention in patients with advanced dementia.

Consistent with this interpretation, Girard et al. reported that although visual acuity improved after cataract surgery in patients with moderate dementia, no significant improvement was observed in overall neuropsychiatric symptoms, as measured by the neuropsychiatric index (NPI) [19].

Notably, while sleep disturbances improved postoperatively, agitation increased significantly, suggesting that visual restoration may be associated with increased patient activity rather than global behavioral stabilization. In the present study, the persistence of caregiver burden despite visual improvement may similarly reflect increased patient activity following visual recovery, resulting in sustained supervision and caregiving demands.

Furthermore, recent findings have demonstrated that cataract surgery improves visual function and brain activity patterns related to delirium in older adults with higher baseline cognitive function (median MMSE: 28.0) [20]. However, such physiological improvements may not be sufficient to address the complex behavioral and functional challenges in moderate-to-severe dementia. The present findings do not address the effects of intervention timing. Nevertheless, they generate the hypothesis that visual interventions may have different effects on caregiver outcomes if implemented earlier in the course of cognitive decline. This hypothesis remains speculative and warrants investigation in future prospective studies.

Our findings are further corroborated by the recent large-scale SENSE-Cog randomized controlled trial, which reported that a comprehensive, 18-week sensory support intervention—including the provision of hearing aids and glasses—failed to improve health-related QoL in patients with mild-to-moderate dementia [21]. The lack of significant impact in their well-powered multicenter study reinforces our observation that correcting sensory deficits alone may be insufficient to modify established psychosocial outcomes in dementia care. Taking together, these results suggest that for patients already living with established cognitive decline, neither a single-domain intervention like cataract surgery nor a multidisciplinary sensory rehabilitation program will lead to immediate measurable shifts in complex outcomes such as caregiver burden or patient QoL.

Furthermore, the methodological challenges encountered in this study reflect the broader landscape of sensory intervention research in advanced dementia. The recent SENSE-Cog Residential Care (SSI-RC) project was designed as a cluster-randomized feasibility trial, highlighting the practical and ethical complexities inherent in this population [22]. In individuals with established dementia, obtaining informed consent, maintaining adherence, and ensuring longitudinal follow-up are often challenging due to cognitive impairment, comorbidities, and high attrition. In this context, the relatively high retention rate observed in the present study provides supportive evidence that follow-up of caregiver burden is feasible. These findings contribute to the evidence base required to inform the design of future definitive randomized controlled trials.

While these findings highlight potential mechanisms underlying the persistence of caregiver burden, much of the existing literature on ophthalmic surgery in older patients with dementia has focused primarily on patient-centered outcomes, such as surgical safety, perioperative risks, and adverse events.

In parallel, several studies have examined patient-related outcomes, particularly whether improvements in visual function may contribute to cognitive function and QoL [7]. In our previous study, we identified clinical factors associated with adverse outcomes following ophthalmic surgery in older patients with dementia, underscoring the importance of careful patient selection and meticulous perioperative management in this vulnerable population [23].

Building upon this patient-centered literature, the present study broadens the focus to caregiver-centered outcomes. While patient-level risks and benefits remain critical considerations, our findings suggest that improvements in visual function do not necessarily translate into short-term reductions in caregiver burden once dementia and caregiving needs are established. This discrepancy highlights the need to evaluate ophthalmic interventions in patients with dementia not only in terms of surgical safety and visual outcomes, but also from the perspective of caregivers who support patients in their daily lives, given that caregiver well-being is essential for the long-term sustainability of home-based dementia care. This finding is consistent with previous literature identifying caregiver age as an important factor in adjustment to vision loss. For example, Bambara et al. reported that caregivers at risk for depression were significantly younger than those not at risk [24], suggesting that younger caregivers may experience distinct psychosocial stressors and differ from older caregivers in their coping strategies for managing caregiving-related challenges.

Our exploratory findings further support the vulnerability of younger caregivers in the context of visual rehabilitation. One possible explanation is the concept of developmental burden, as described in a recent multicenter study by Virgili et al. [25]. Developmental burden reflects caregivers’ perceptions of being disconnected from age-appropriate social, professional, or life-course opportunities due to caregiving responsibilities. Younger caregivers, often adult children balancing employment and childcare, may experience this form of role conflict more acutely. As a result, even when patients’ visual acuity improves following surgery, these caregivers may be less likely to perceive a meaningful reduction in overall burden. However, this interpretation remains speculative and should be confirmed in larger prospective studies. In addition, residual confounding and differences in caregiver characteristics or caregiving roles may also have contributed to this association.

Furthermore, despite significant improvement in visual function, no detectable short-term improvement was observed in cognitive function or caregiver burden. These findings suggest that restoration of visual function alone may not be sufficient to produce measurable short-term changes in caregiver burden among patients with established dementia. It is important to acknowledge that caregiver burden is a multidimensional outcome and that key caregiver-related factors—including caregiver role, hours of care, co-residence status, employment status, and psychological background—were not systematically collected in this study. These unmeasured variables may have influenced caregiver burden independently of visual function and should therefore be considered when interpreting the primary outcome.

A recent pilot study of a sensory intervention in people with dementia primarily focused on feasibility outcomes, including recruitment, retention, and adherence, rather than definitive efficacy [22]. This reflects the practical and methodological challenges of conducting large-scale randomized controlled trials in this vulnerable population. In this context, the present study provides complementary observational evidence, demonstrating the feasibility of postoperative follow-up and offering preliminary estimates of effect sizes to inform the design of future interventional studies.

Several limitations should be considered when interpreting the findings of this study.

First, this was a single-center study involving a relatively small sample of patients who were considered suitable candidates for cataract surgery and agreed to participate. Therefore, selection bias cannot be excluded, and the generalizability of the findings to other clinical settings or more medically complex populations may be limited.

Furthermore, the single-arm pre-post design without a control group significantly limits the causal interpretation of our findings. We cannot separate the effect of surgery from natural changes over time, caregiver expectations, or other changes in the care environment. Therefore, this study provides preliminary observational data rather than definitive evidence of a causal effect on caregiver burden.

Second, the sample size was relatively small (*n* = 28), reducing the statistical power to detect smaller effects and underscoring the preliminary nature of the findings. Although the retention rate was high (84.8%), the possibility of attrition bias resulting from the loss to follow-up of five patient–caregiver pairs should also be considered. Because of the small number of non-completers, formal comparison between completers and non-completers was not feasible.

Third, the study population consisted predominantly of patients with moderate-to-severe dementia; therefore, the results may not be generalizable to individuals with milder cognitive impairment or those at earlier stages of the dementia continuum. In addition, the follow-up period was relatively short, and longer-term effects of visual improvement on caregiver burden cannot be excluded.

Although the MMSE and BI are widely used and clinically practical measures, more comprehensive assessments may provide additional information regarding cognitive and functional outcomes in future studies.

Fourth, caregiver burden was assessed using the caregiver-reported J-ZBI, which is inherently subjective and may have been influenced by individual perceptions and psychosocial factors. Furthermore, we did not quantify caregiving intensity, such as the number of hours devoted to caregiving, nor did we assess pre-existing psychiatric conditions among caregivers, both of which may influence the perception and reporting of caregiver burden, as suggested in previous studies [24].

In addition, the majority of caregivers in the present study were adult children (75%), with relatively few spousal caregivers included. Previous studies have suggested that perceived caregiver burden may vary according to the caregiver–patient relationship, as caregiving experiences and role expectations can differ between adult children and spouses [25]. The predominance of adult-child caregivers in this sample may therefore have influenced the observed J-ZBI scores and may limit the generalizability of our findings.

Finally, baseline physical comorbidities including severe motor disability and physical frailty were not specifically categorized or adjusted for in the analysis. Although functional status was assessed using BI, these conditions may independently limit activities of daily living and confound the relationship between visual improvement and changes in caregiver burden.

Consequently, residual confounding cannot be excluded. Future larger prospective studies should incorporate more comprehensive assessments of both patient- and caregiver-related factors to better isolate the effects of visual restoration on caregiver outcomes.

## 5. Conclusions

In this prospective pilot study, cataract surgery effectively improved visual acuity in older patients with moderate-to-severe dementia. However, this visual improvement did not translate into detectable short-term reductions in established caregiver burden. The high retention rate (84.8%) demonstrates the feasibility of conducting longitudinal follow-up and delivering surgical interventions in this challenging population.

Our findings suggest that although cataract surgery improves visual function in patients with advanced dementia, restoration of visual function alone may not be sufficient to produce measurable short-term reductions in caregiver burden over a 3-month follow-up period. Therefore, while clinically beneficial for vision restoration, cataract surgery should be integrated into a broader multidisciplinary care approach. These preliminary findings provide a foundation for future larger-scale prospective studies to examine whether the timing of visual intervention may influences caregiver outcomes across different stages of cognitive impairment.

### Clinical Implications

- Cataract surgery effectively improves visual function in patients with dementia but may not immediately reduce caregiver burden over a short-term follow-up period.
- Future studies should investigate whether the timing of visual intervention influences caregiver outcomes across different stages of cognitive impairment.
- Caregiver-related factors should be considered when interpreting caregiver burden, particularly because caregiver characteristics and caregiving context may influence postoperative outcomes.

## Figures and Tables

**Table 1:** Baseline characteristics of patients with dementia and their caregivers (*n* = 28).

Characteristic	Mean ± SD or *n* (%)	Range
Patients		
Age (years)	85.5 ± 5.7	75–98
Sex (Male/Female)	7 (25%)/21 (75%)	-
MMSE score	13.1 ± 5.6	0–22
Visual acuity (logMAR)	0.70 ± 0.65	−0.08–2.0
Barthel Index	72.3 ± 20.3	35–100
Anesthesia (General/Local)	10 (35.7%)/18 (64.3%)	
Surgical laterality (Bilateral/Unilateral)	22 (78.6%)/6 (21.4%)	
Caregivers		
Age (years)	59.0 ± 12.3	42–85
Relationship to patient		
Spouse	6 (21.4%)	-
Child	21 (75.0%)	-
Grandchild	1 (3.6%)	-

SD, Standard Deviation; MMSE, Mini-Mental State Examination. Baseline demographic and clinical characteristics of older patients with dementia undergoing cataract surgery and their primary caregivers. Continuous variables are presented as mean ± standard deviation, and categorical variables are presented as number (%).

**Table 2:** Comparison of clinical outcomes at baseline and 3 months after cataract surgery.

Variable	Preoperative	Postoperative	95% CI	*p*-Value
Visual acuity (logMAR)	0.70 ± 0.65	0.11 ± 0.24	0.37 to 0.82	<0.001
Caregiver Burden (J-ZBI)	29.9 ± 15.0	27.8 ± 13.2	−3.91 to 8.05	0.48
ADL (Barthel Index)	72.3 ± 20.3	75.2 ± 18.6	−6.82 to 1.11	0.15
Cognitive Score (MMSE)	13.1 ± 5.6	13.2 ± 6.2	−1.68 to 1.47	0.89

J-ZBI, Japanese version of the Zarit Burden Interview; MMSE, Mini-Mental State Examination; CI, confidence interval.

**Table 3:** Exploratory multivariable linear regression analysis of factors associated with postoperative caregiver burden.

Variable	Coefficient (β)	95% CI	*p*-Value
Preoperative J-ZBI score	0.44	0.12 to 0.75	0.008
Caregiver age	−0.38	−0.76 to −0.006	0.047

β; Standardized regression coefficients, CI; confidence interval, J-ZBI; Japanese version of the Zarit Burden Interview.

## Data Availability

The datasets used and analyzed during the current study are available from the corresponding author upon reasonable request, subject to approval by the institutional ethics committee.

## Abbreviations

BCVA	Best-Corrected Visual Acuity
BI	Barthel Index
JTKGMC	Juntendo Tokyo Koto Geriatric Medical Center
J-ZBI	Japanese version of the Zarit Burden Interview
MCI	Mild Cognitive Impairment
MMSE	Mini-Mental State Examination
QoL	Quality of Life
ZBI	Zarit Burden Interview
